# Sepsis in the Structurally-Vulnerable Heart: A Case of Infective Endocarditis Masquerading as a Urinary Tract Infection in Pediatric Rheumatic Heart Disease

**DOI:** 10.7759/cureus.94673

**Published:** 2025-10-15

**Authors:** Anna Mikami, Michael Sherwood, Abrag Nassar, Jennifer Burnham, Miriam Benavides, Samhrutha Sripathi, Chezhiyan Murugesan

**Affiliations:** 1 Pediatrics, Kern Medical Center, Bakersfield, USA

**Keywords:** infective endocarditis, janeway lesion, pediatric sepsis, persistent hypotension, pyelonephritis, rheumatic heart disease (rhd)

## Abstract

We present a case of a six-year-old female patient with a history of rheumatic heart disease (RHD), including mitral stenosis, mitral regurgitation, and pulmonary hypertension, who presented with fever, abdominal pain, and urinary findings. Although urinalysis and imaging confirmed bilateral pyelonephritis, the admitting provider noted concerning physical exam findings, including Janeway lesions and systemic features that raised suspicion for concurrent infective endocarditis (IE). In the context of her underlying valvular disease, these exam clues broadened the differential beyond a presumed urinary source and prompted an endocarditis-focused evaluation. This case underscores the enduring value of a careful physical examination, the need to maintain a wide diagnostic lens, and the importance of tailoring sepsis management for pediatric patients with complex cardiac comorbidities.

## Introduction

Rheumatic heart disease (RHD) is a chronic sequela of repeated episodes of acute rheumatic fever, leading to progressive damage of the heart valves [1]. Globally, RHD affects an estimated 40 million individuals and is responsible for approximately 500,000 deaths annually, with the vast majority occurring in low- and middle-income countries [2]. In developed nations, the incidence and prevalence of RHD have markedly declined, primarily due to the widespread use of antibiotics to treat Group A Streptococcal infections and overall improvements in living conditions [3].

As a result, it is now uncommon for children in developed countries to present with infective endocarditis (IE) in the setting of pre-existing RHD [4]. However, individuals with RHD remain at elevated risk for IE due to turbulent blood flow, which causes endothelial trauma, deposition of platelets and fibrin on the endothelium, and an opportunity for invading bacteria to colonize these deposits [5]. The majority of IE cases are caused by gram-positive organisms, particularly staphylococci, streptococci, and enterococci [6].

Bacteremia in pediatric patients with RHD may originate from various sources, with urinary tract infections (UTIs), including pyelonephritis, being among the most common. This overlap in infectious etiologies presents significant diagnostic and management challenges. Clinical features such as fever and elevated inflammatory markers may be attributed solely to a UTI, thereby delaying recognition of IE. Conversely, bacteremia secondary to a UTI can seed damaged valves and precipitate IE [5].

This case report explores the interplay between concurrent UTI/pyelonephritis and IE in a child with underlying RHD, highlighting the diagnostic complexities, clinical decision-making, and outcomes based on current case literature and practice guidelines.

## Case presentation

A six-year-old female patient with a history of RHD complicated by mitral regurgitation, mitral stenosis, dysplastic aortic valve, and pulmonary hypertension, managed with monthly penicillin injections (recently noncompliant), enalapril, furosemide, and spironolactone, presented to the emergency department with four days of poor oral intake, decreased urine output, abdominal pain, fever, and right leg pain.

**Figure 1:**
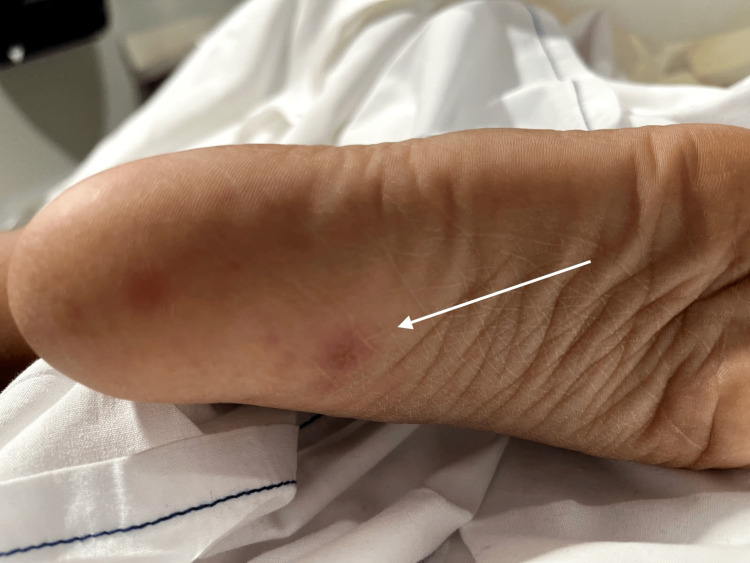
Janeway lesions on the right foot

On presentation, she was febrile to 39.5ºC, tachycardic, and tachypneic. Physical examination revealed right lower quadrant abdominal tenderness, a cardiac murmur, Janeway lesions on both feet, and tenderness of the right thigh (Figure 1).

Initial labs were notable for leukocytosis (white blood cells (WBC) 16,000 x 10^9^/L) with a left shift (69% segmented neutrophils, 5% bands), elevated CRP (16.7 mg/dL), lipase 152 U/L, and a normal lactic acid (1.6 mmol/L) (Table 1).

**Table 1:** Labs for hospital day one to four with normal ranges and units of measurement WBC, white blood cells; CRP, C-reactive protein; AST, aspartate transaminase; L, liter; mg/L, milligrams/liter; mmol/L, millimoles/liter; ng/mL, nanograms/milliliters; U/L, units/liter

Hospital Day (HD)	WBC (3.8-10.4 x 10^9^/L)	CRP (<10 mg/L)	Lactic acid (0.4-2.0 mmol/L)	Procalcitonin (<=0.15 ng/mL)	Lipase (13-75 U/L)	AST (15-37 U/L)	Blood cultures (Negative)
HD 1	16	16.7	1.6	22.56	152	45	
HD 2	18	19	-	-	-	-	Staph aureus
HD 3	16.9	15.4	1.21	-	-	-	-
HD 4	20.1	14.1	1.15	-	-	-	Staph aureus

**Figure 2:**
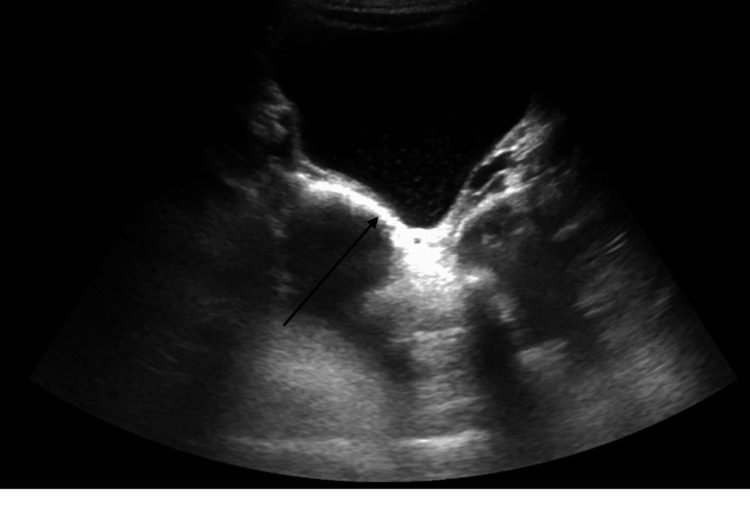
Ultrasound of the urinary bladder showing specular debris

A viral respiratory pathogen panel was negative. Abdominal ultrasound showed specular debris within the urinary bladder (Figure 2).

**Figure 3:**
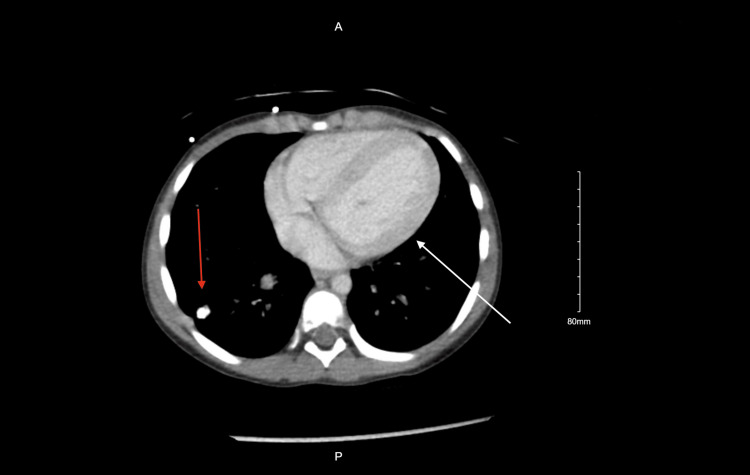
Contrast enhanced CT of the chest showing mild cardiomegaly and left ventricular dilation (white arrow) as well as a right lung base granuloma (red arrow)

**Figure 4:**
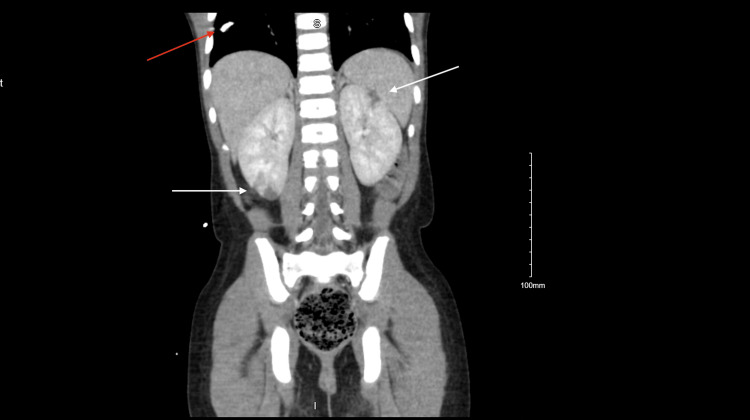
Contrast enhanced CT of the abdomen and pelvis showing right lung base granuloma (red arrow), and bilateral pyelonephritis with multifocal perfusion defects (white arrows)

A contrast-enhanced CT of the abdomen and pelvis demonstrated bilateral pyelonephritis with multifocal perfusion defects, nonspecific bladder distension, right lung base calcified granuloma, mild cardiomegaly, and left ventricular dilation (Figures 3, 4).

Given the concern for sepsis, the patient was started on 1 gram of ceftriaxone initially followed by empiric IV antibiotics (Zosyn and vancomycin) and received 5 cc/kg of IV fluid bolus secondary to hypotension and at a reduced rate due to her cardiac history. X-rays of the bilateral femurs were obtained for right thigh pain and were unremarkable. Blood cultures later grew gram-positive cocci resembling Staphylococcus. The patient had multiple episodes of hypotension, therefore further workup was obtained to evaluate for other sources of infection in light of the patient’s physical exam findings. Repeat blood cultures were obtained and an echocardiogram showed severe mitral insufficiency with mild mitral stenosis, a dysplastic aortic valve, moderately dilated left atrium, and mildly enlarged left ventricle with preserved ventricular function and no pericardial effusion, but was unable to identify obvious vegetations.

Due to the concern for bacteremia with potential bilateral renal infarcts from septic emboli, likely secondary to IE, and the complexity of fluid management in the context of underlying cardiac disease, the patient was transferred to a higher-level care facility for Pediatric Intensive Care Unit (PICU) admission. Another set of blood cultures were obtained and a repeat echocardiogram demonstrated mildly worsened severe mitral insufficiency, moderate mitral stenosis, and thickened mitral valve leaflets. We were unable to rule out endocarditis. The patient was diagnosed with endocarditis as she met one major clinical criteria and four minor clinical criteria of the Duke Criteria [7]. 

All final blood cultures grew pan-sensitive methicillin-sensitive Staphylococcus aureus (MSSA). The antibiotic therapy was changed to vancomycin and cefazolin (Ancef) as per infectious disease recommendations, based both on institutional protocol and provider practice. A peripherally inserted central catheter (PICC) line was placed and antibiotic therapy was then tailored to just cefazolin following the positive blood culture sensitivities. Therefore, the patient was on six weeks of cefazolin therapy alone from the first set of negative blood cultures.

She was discharged on her home cardiac medications (furosemide, spironolactone, and enalapril), with instructions to take prophylactic antibiotics (Keflex) prior to any future dental procedures in addition to her prophylactic penicillin. 

## Discussion

This six-year-old female patient with preexisting RHD initially presented with fever and flank/abdominal pain, findings suggestive of a urinary tract infection, supported by imaging consistent with bilateral pyelonephritis. However, persistent hypotension and further physical examination revealed bilateral pedal Janeway lesions, raising concern for concurrent IE and concern for hypovolemic versus cardiogenic shock.

The presence of a UTI could easily lead to anchoring bias, attributing systemic signs such as fever, leukocytosis, and elevated CRP solely to the UTI. This is a common pitfall in misdiagnosed IE cases with urinary tract pathologies [8,9]. Nevertheless, several features in this patient, including recurrent episodes of hypotension, a known heart condition, murmur, Janeway lesions, and high fever, heightened suspicion for IE, especially in the context of known valvular pathology.

This scenario mirrors previous case reports in which pyelonephritis masked underlying IE, with the diagnosis delayed until embolic phenomena or persistent symptoms prompted further investigation [10]. In this case, persistent hypotension, as well as early recognition of Janeway lesions and auscultatory findings, likely helped sustain momentum toward appropriate evaluation, including echocardiography.

The initial use of ceftriaxone followed by broad-spectrum empiric antibiotics, piperacillin and tazobactam (Zosyn) and vancomycin, was clinically appropriate, targeting both gram-negative pathogens associated with UTIs and gram-positive organisms (including Staphylococcus aureus and enterococci) implicated in IE [11]. Notably, the care team administered a conservative fluid bolus (5 cc/kg), reflecting awareness of the patient’s hemodynamic vulnerability due to mitral stenosis and pulmonary hypertension. This fluid-sparing approach aligns with the current literature emphasizing cautious resuscitation in patients with significant valvular disease or right-sided pressure overload, where aggressive fluid administration can exacerbate pulmonary edema or trigger right heart failure [12].

Although the abdominal and pelvic CT was obtained to investigate abdominal pain, it provided additional diagnostic value by revealing mild cardiomegaly and left ventricular dilation, potential indicators of decompensated cardiac function or evolving sequelae of IE. The identification of a calcified granuloma at the right lung base, though likely incidental, warranted a more comprehensive infectious disease evaluation, particularly in a febrile pediatric patient.

This case underscores the need for high clinical vigilance when evaluating febrile children with structural heart disease. The presence of a confirmed UTI should not exclude the possibility of a concurrent, more serious pathology such as IE. The overlap in systemic inflammatory markers and symptoms between these infections can obscure the true diagnosis, delaying treatment and increasing the risk of complications such as embolic events and prolonged hospitalization. It reinforces findings in the literature that coexisting infections can increase morbidity, and that timely diagnosis and intervention in pediatric IE are critical to optimizing outcomes.

## Conclusions

This case relays the importance of maintaining a broad differential when evaluating febrile illness in pediatric patients with preexisting structural heart disease. While UTIs are common and often the presumed source of infection in children with abdominal pain and fever, this case illustrates how coexisting pathologies, such as IE, can be masked by more readily apparent diagnoses like pyelonephritis.

Prompt recognition of clinical red flags, including Janeway lesions, a cardiac murmur, and systemic signs of embolization, was critical in initiating appropriate early management. The use of broad-spectrum empiric antibiotics and fluid resuscitation tailored to the patient's cardiac physiology demonstrated appropriate modification of standard protocols in the context of comorbid rheumatic valvular disease and pulmonary hypertension.

Ultimately, this case highlights the need for multidisciplinary care, early cardiac imaging, and heightened vigilance for infective endocarditis in patients with known valvular abnormalities, even in the setting of a seemingly isolated urinary infection. Timely diagnosis and individualized management are essential to optimizing outcomes and preventing the significant morbidity associated with delayed treatment in this vulnerable population.
